# Potential risk of porcine-derived pancreatic enzyme medication for the cross-species transmission of hepatitis E virus

**DOI:** 10.1136/gutjnl-2024-332577

**Published:** 2024-05-31

**Authors:** Nicola Frericks, Volker Kinast, Eike Steinmann

**Affiliations:** 1 Department of Molecular and Medical Virology, Ruhr University Bochum, Bochum, Germany; 2 Department of Medical Microbiology and Virology, Carl von Ossietzky Universität Oldenburg, Oldenburg, Germany; 3 German Centre for Infection Research (DZIF), External Partner Site, Bochum, Germany

**Keywords:** HEPATITIS E, CYSTIC FIBROSIS

Infection with the hepatitis E virus (HEV) is the leading cause of acute viral hepatitis worldwide. With an estimated number of 20 million infections and 3.3 million symptomatic cases, accounting for 3.3% of viral hepatitis-related deaths annually, HEV poses a major public health threat.^1 2^ Transmission of HEV occurs mainly by the faecal-oral route in low-income and middle-income countries due to poor sanitation. Here, endemic outbreaks and sporadic cases are mainly caused by infection with genotypes 1 and 2 (HEV-1 and HEV-2), which exclusively infect humans. In developed countries, only sporadic cases of HEV infection are reported, which are attributed mostly to zoonotic genotypes 3 and 4 (HEV-3 and HEV-4) infections. As swine represents the main reservoir for HEV-3, transmission is usually linked to the consumption of undercooked pork products.^3^ However, due to the lack of specific antiviral therapy, thorough risk assessment is crucial to protect vulnerable populations. Especially solid organ transplant recipients are a high-risk population, since HEV-3 infections in immunocompromised individuals are frequently associated with a chronic course of disease and fast progression to cirrhosis.^3^ Although lung transplant recipients receive strong immunosuppressive therapy, research on chronic hepatitis E (CHE) in this population is limited due to the low number of reported cases.^4^

Lung transplantation is a surgical measure for patients with end-stage lung disease caused by cystic fibrosis (CF). In fact, patients with CF (pwCF) account for 15% of adult lung transplant recipients worldwide.^5^ Although primarily damaging the lungs, the impairment of mucus hydration and clearance caused by the hereditable multisystemic disease also leads to pancreatic autodigestion and insufficiency in approximately 80% of pwCF.^6^

**Figure 1:**
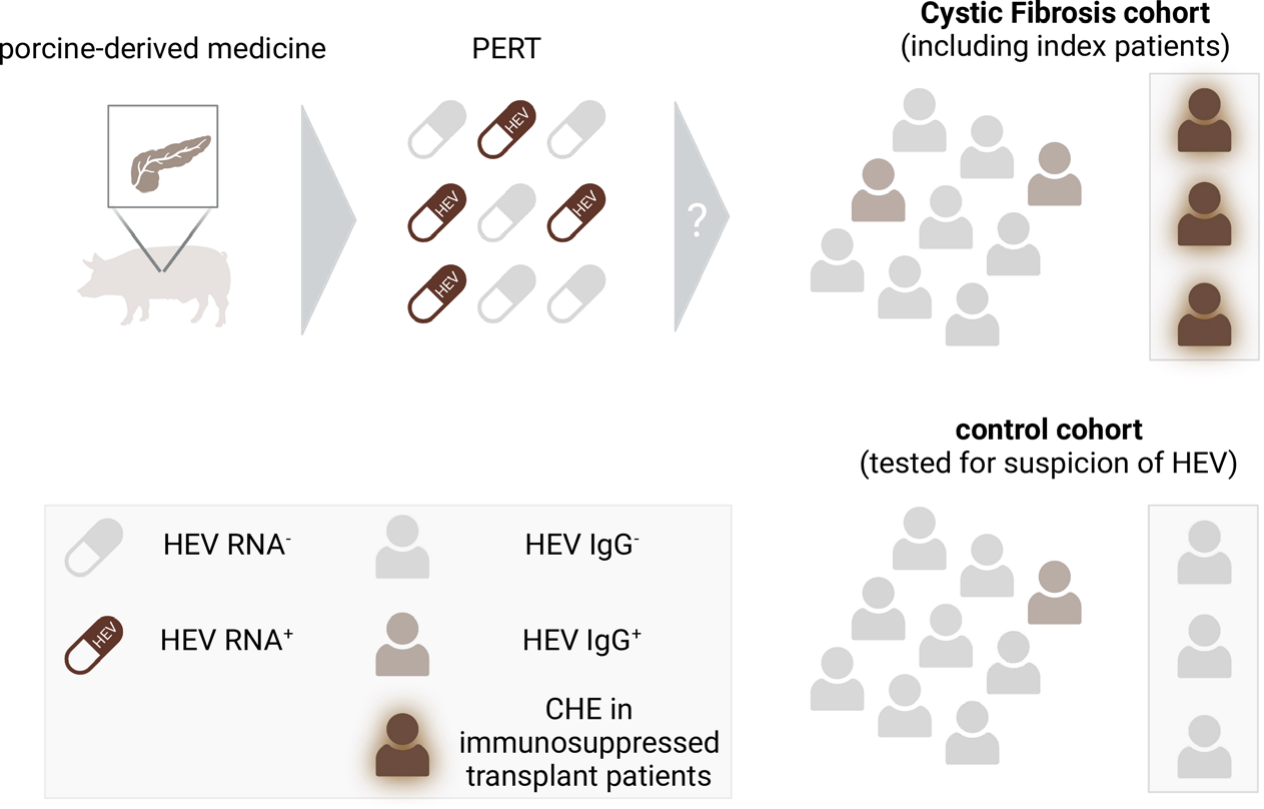
High prevalence of HEV-3 RNA in PERT correlates with an increased HEV seroprevalence in a Canadian cohort of individuals suffering from cystic fibrosis. Roughly 40% of porcine-derived PERT medications sourced from Canadian suppliers exhibited detectable levels of HEV-3 RNA. The potential contamination of PERT with infectious HEV might contribute to the increased prevalence of HEV antibodies and the chronic progression of HEV infection among cystic fibrosis patients when compared with a control group showing clinical indications of viral hepatitis. Figure created with BioRender.com. HEV, hepatitis E virus; PERT, pancreatic replacement therapy.

In *Gut*, Thornton *et al* 7 diagnosed the first three cases of CHE, which were associated with CF-induced lung transplantation in Canada (figure 1). As these are three of four CHE cases ever described in Canada, they assessed the HEV seroprevalence of all pwCF in a regional clinical centre (Southern Alberta Adult CF Clinic), regardless of transplant status, to elucidate the causative factors behind the cluster of CHE. A cohort of individuals with clinical indications for viral hepatitis served as a control group. Interestingly, the HEV seropositivity among pwCF, regardless of transplant status, was twofold higher compared with the cohort with a clinical suspicion for HEV infection and was fourfold higher than in the general Canadian population. While neither administration of blood transfusion nor demographic origin or consumption of undercooked or uncommon pork products were associated with the diagnosis of CHE, Thornton *et al* identified a unique feature of pwCF—the routinely administered pancreatic replacement therapy (PERT) to treat pancreatic insufficiency of pwCF. Since PERT consists of pancreatic enzymes extracted from swine, the authors hypothesised that this porcine-derived medication could be a plausible source of the observed disproportionate HEV seropositivity within the pwCF population.

Indeed, by screening for the presence of viral traces in PERT medication, the authors were able to show that more than 40% of PERT capsules from all four manufacturers approved in Canada were positive for HEV *orf1* and *orf2* RNA genomic regions. Sequencing of patient samples and viral RNA extracted from PERT capsules revealed a close phylogenetic relationship to previously identified human-derived and swine-derived HEV-3 isolates. Although the median value of 50 HEV copies per capsule appears to be quite low, and numerous efforts by the authors to overcome experimental challenges to proove the presence of infectious virus were unsuccessful, it has to be acknowledged that the assessment of 107 capsules for infectious HEV was probably underpowered as a single patient consumes in median about 9000 capsules per year.^7^ Given that HEV exhibits high thermal and surface stability,^8^ infectious viruses might persist throughout capsule manufacturing. Moreover, the infection rate of the pancreas is notably high in swine,^9^ resulting in an initial viral load within the primary tissue, likely at a level high enough for infectious virus to persist. To the best of our knowledge, no viral transmission to humans upon treatment with porcine-derived medications has been reported so far. However, the risk of zoonotic transmission of HEV via PERT administration has been discussed previously.^10^ In general, the risk of viral transmission by porcine-derived medications might not be limited to HEV or PERT: transmission of other viruses with human and swine tropism (ie, Japanese encephalitis virus, Nipah virus and swine/avian influenza strains) should also be considered as a risk factor for other vulnerable patient groups.

Since the observations by Thornton *et al* are based only on one single-centre study, the findings should be confirmed also in other CF and transplant centres. Although a causal relation between the high prevalence of HEV RNA in PERT and the high seropositivity in pwCF remains to be established, Thornton *et al* provide the first evidence of the risk of viral transmission from porcine-derived pancreatic enzyme medications. In our opinion, this study should be seen as an encouragement to further investigate HEV seroprevalence in other pwCF cohorts and ultimately raise the awareness of a potential risk of iatrogenic HEV acquisition through animal-derived medications in general, as the cure from CHE remains tremendously challenging until today.
